# A Study on the Characteristics and Outcomes of Reported Diphtheria Patients in a Western State in India

**DOI:** 10.7759/cureus.35769

**Published:** 2023-03-04

**Authors:** Sadab Boghani, Harsh D Shah, Manish Fancy, Trushar Parmar, Shikha Bansal, Mayur B Wanjari, Deepak Saxena

**Affiliations:** 1 Department of Public Health Science, Indian Institute of Public Health Gandhinagar, Gandhinagar, IND; 2 Department of Public Health, Health and Family Welfare, Government of Gujarat, Gujarat, IND; 3 Department of Public Health, Management Sciences for Health, New Delhi, IND; 4 Department of Public Health, World Health Organization, New Delhi, IND; 5 Department of Research and Development, Jawaharlal Nehru Medical College, Datta Meghe Institute of Medical Sciences, Wardha, IND

**Keywords:** respiratory mucosa and skin, disease surveillance, immunization, vaccine-preventable diseases, diphtheria

## Abstract

Background

The incidence of diphtheria cases has declined significantly from 1,00,000 cases in 1980 to 2500 in 2015 globally. India contributed to half of the diphtheria cases reported globally from 2001 to 2015. The disease has higher case mortality and morbidity rate due to various geographic-specific factors. The current study aims to outline the characteristics and outcomes of the diphtheria-reported patients of Gujarat, a western state of India.

Method

A record-based, descriptive retrospective study was undertaken in the western state of India by analyzing district-wise reported diphtheria cases in diphtheria, tetanus, and pertussis (DPT) surveillance program format during 2020-2021.

Result

Out of 446, most patients were reported from selected geographies of Gujarat state in 2020-2021. The 424 (95%) reported cases were from 0-14 years of age. Only 9 (2%) subjects had a travel history, and 369 (82.7%) patients were reported from rural areas. The time trend analysis showed that 339 (76%) patients were reported from September to December. The case-fatality ratio was 5.4%, and 300 (67.2%) cases didn’t take the DPT (DPT3)/pentavalent 3rd dose vaccine and subsequent doses during their lifetime, emphasizing the role of the vaccine in preventing diphtheria disease.

Conclusion

Increased vaccination coverage and completing all doses of the DPT vaccine are crucial to avert deaths due to diphtheria. An effective surveillance system will aid in early disease detection and provide more information on the factors that lead to disease occurrence for prompt action by the authority.

## Introduction

Infection with vaccine-preventable diseases (VPD) can cause life-threatening episodes, including deaths [1]. Diphtheria, a VPD, is caused by the toxin-producing gram-positive bacterium, *Corynebacterium diphtheria*. It mainly affects the respiratory mucosa and skin, causing respiratory diphtheria and cutaneous diphtheria. It transmits from person to person by respiratory droplets or contacting respiratory secretions, discharges from a skin lesion, or rarely fomites [2].

With the introduction of vaccines and adoption of the diphtheria vaccine in universal immunization programs by various national governments, morbidity and mortality of diphtheria have significantly reduced [3-6]. Diphtheria, pertussis, and tetanus (DPT) surveillance is part of a Universal Immunization Program (UIP) as per the DPT surveillance guidelines issued by the Ministry of Health and Family Welfare (MoHFW), New Delhi, Government of India. Under the UIP national program, the DPT vaccine has been replaced with pentavalent (DPwT-HBV-Hib) for infants with schedules of 6 weeks (first dose), ten weeks (second dose), and 14 weeks (third dose) after birth since 2012. The diphtheria vaccine is given at 6, 10, and 14 weeks of age; the diphtheria booster dose is between 16-24th months (first booster) and 5-6 years (second booster), and the Td is given at the age of 10 and 16 years of age and to the pregnant women [7-9]. Per the National Family Health Survey (NFHS) survey, diphtheria 3 dose vaccine coverage has increased significantly between 1992 and 1993 (NFHS -1) and 2019 and 2020 (NFHS -5) in India (from 51.7% in NFHS 1 to 86.7% in NFHS 5 ) and Gujarat (from 63.8% in NFHS 1 to 86.1% in NFHS 5), contributing to the decline in diphtheria cases over time [3-5].

The incidence of diphtheria cases has declined significantly from 1,00,000 cases in 1980 to 2500 cases in 2015 globally. India contributed to half of the diphtheria cases reported globally from 2001 to 2015 [3]. India registered an average of 4167 cases and 92 deaths annually from 2005 to 2014. In 2018, India reported 8788 cases of diphtheria to WHO and United Nations Children’s Fund through the Joint Reporting Form. The disease fatality rate of diphtheria is 5-10 percent, with higher death rates of up to 20% among children under five years and individuals older than 40 years of age [4]. Diseases have been completely eradicated from many advanced economies due to improved vaccination status, pediatric care, and hygiene status. However, concern remains among developing countries due to inadequate vaccination coverage and training of health staff in identifying and treating diphtheria cases [5]. Several factors influence the incidence of diphtheria, like low vaccination status, crowding, migration, nutritional status and personal hygiene of children, house ventilation, and humidity in the house [6]. Many studies have been undertaken to understand the factors leading to the outbreak of diphtheria but in a limited geographical area [7,8]. The current study aims to identify various socio-economic, demographic, geographical, and programmatic factors contributing to the occurrence of diphtheria in Gujarat, a western state of India, through descriptive analysis of DPT surveillance data.

## Materials and methods

Study design

A descriptive retrospective study analyzed VPD data reported in DPT surveillance systems. The DPT surveillance is part of a UIP per the DPT surveillance guidelines issued by the MoHFW, New Delhi, Government of India [9].

Study area

The study was conducted in the western state of India, Gujarat, with a population of 60.4 million. The State has 33 districts and eight corporations as a reporting unit on different national health programs. In the district, the District Immunization Officer, headed by the Chief District Health Officer, oversees the surveillance and response system. The district is further divided into the sub-district, i.e., the Taluka, whose overall charge is the Taluka Medical Officer.

Study population

The study included all the reported diphtheria cases received in the DPT surveillance format between March and April 2020-2021. The cases were notified as diphtheria through the DPT surveillance program as per the national guideline.

Data collection and data variable

The DPT surveillance data is reported to districts from health center units of the districts and further to the state every week. These centers are categorized as the reporting units (medical college hospitals, district hospitals, government health facilities, and private hospitals), the informer units (smaller health facilities or clinics), or via community and active case search when the cases are reported [9].

A total of 446 cases of diphtheria were notified through clinical and laboratory confirmation as per DPT surveillance program guidelines by health units in a specific format during the study period. This information includes the demographic details of the patients, vaccination status, clinical symptoms, contact and travel history, sample investigation, and complication details.

The data was collected from the secondary data reporting format compiled by the office of the state immunization cell, Government of Gujarat. Due care was taken to conceal the identity of the patients during the collection of the report and translated into the study data.

Case definition

The trained staff at the reporting unit made clinical identification of the cases by identifying the patients with symptoms of fever, sore throat, headache, and particularly greyish and whitish discoloration of the throat and tonsils. Also, the nasopharyngeal swab was sent for laboratory confirmation at designated medical colleges’ laboratories [9].

Analysis and statistics

All the data were entered into the excel file (MS Excel 2021) and exported into the statistical software SPSS (IBM Corp. Released 2015. IBM SPSS Statistics for Windows, Version 23.0. Armonk, NY: IBM Corp.). The analysis was based on the frequency and percentage of data variables. The district-wise geographic distribution of diphtheria cases in Gujarat was calculated using the frequency and proportion of data variables. The odds ratio and chi-square test were used to compare the DPT vaccination and survival of the reported case.

Ethical permission and consent

This study aimed to inform the public health response and was carried out as an emergency response to an outbreak. The goal of the study was to advance the public good (beneficence) and social welfare (solidarity); no one was harmed in the process (nonmaleficence); it was fair, honest, and transparent (accountability and transparency); and participant data were de-identified before analysis (confidentiality). The ethical permission sought and received administrative approval from the Department of Health and Family Welfare, Government of Gujarat.

## Results

A total of 446 patients of diphtheria were reported from 14 districts and seven corporations out of 33 districts and eight corporations of Gujarat state in 2020-2021.

Demographic details

Out of 446 patients, 216 (48%) were female, and 230 (52%) were male. The male-female ratio was 1.06 for all reported patients and 1.16 for those without DPT3/pentavalent vaccine. Age-wise distribution showed that the maximum distribution was between 5 and 15 years of age, with 424 (95%) reported from 0-14 years. The reported patients' maximum and minimum age ranges varied from infants to 65 years of age (Table 1). One hundred seventy-three were immunized by the first dose of pentavalent/DPT vaccine, 155 up to the second dose, 144 up to the third dose, 57 first booster dose, and 49 up to the second booster dose out of the 446 patients emphasizing the role of the vaccine in preventing diphtheria. Out of the 446 patients with diphtheria, only 9 (2%) had a travel history, 434 (97%) didn’t have a travel history, and the status of three was unknown. Out of the nine patients with a travel history, one traveled to another state, one to another district, and seven to another block. The average duration of travel was 14 days.

**Table 1:** Age-wise distribution of diphtheria patients (N = 446)

Age (In Years)	Number of diphtheria cases (All)		
	Male	Female	Total
0-04	44 (19.1%)	40 (18.5%)	84 (18.8%)
05-09	116 (50.4%)	102 (47.2%)	218 (48.9%)
10-14	65 (28.3%)	57 (26.4%)	122 (27.4%)
15-19	3 (1.3%)	11 (5.1%)	14 (3.1%)
> 20 years	2 (0.9%)	6 (2.8%)	08 (1.8%)
Total	230 (100%)	216 (100%)	446 (100%)

Geographic distribution

**Figure 1:**
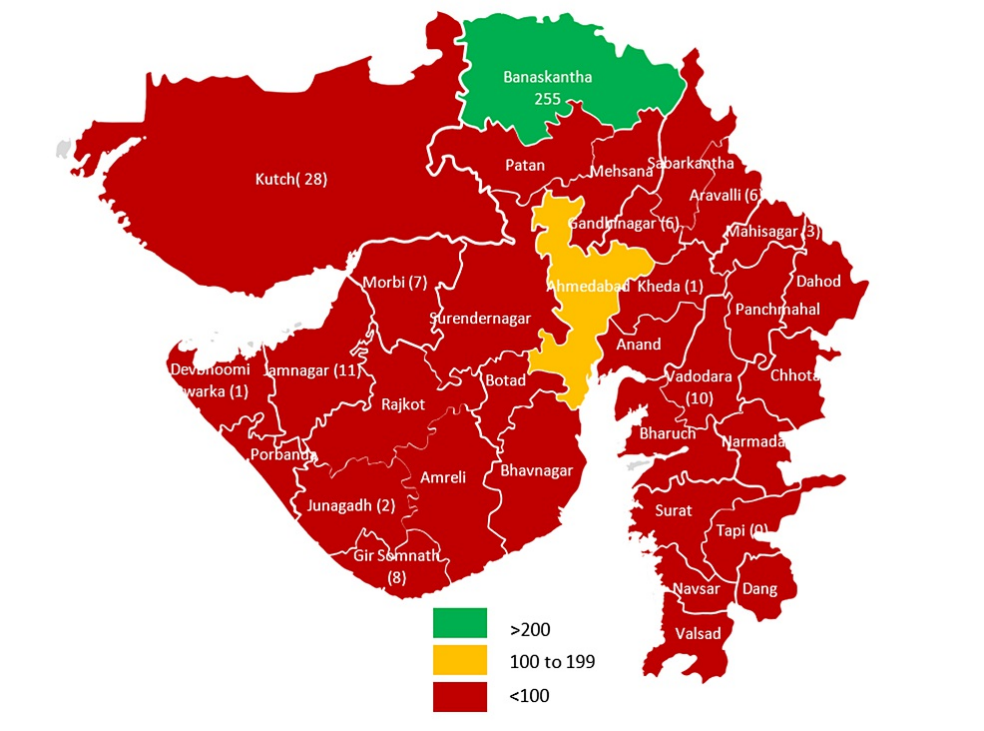
Geographical distribution of diphtheria cases

The majority of the patients were reported from the Banaskantha district 255 (57%), followed by Kutch 28 (6%), Ahmedabad corporation 18 (4%), and Surendranagar 14 (3%) (Figure 1).

**Figure 2:**
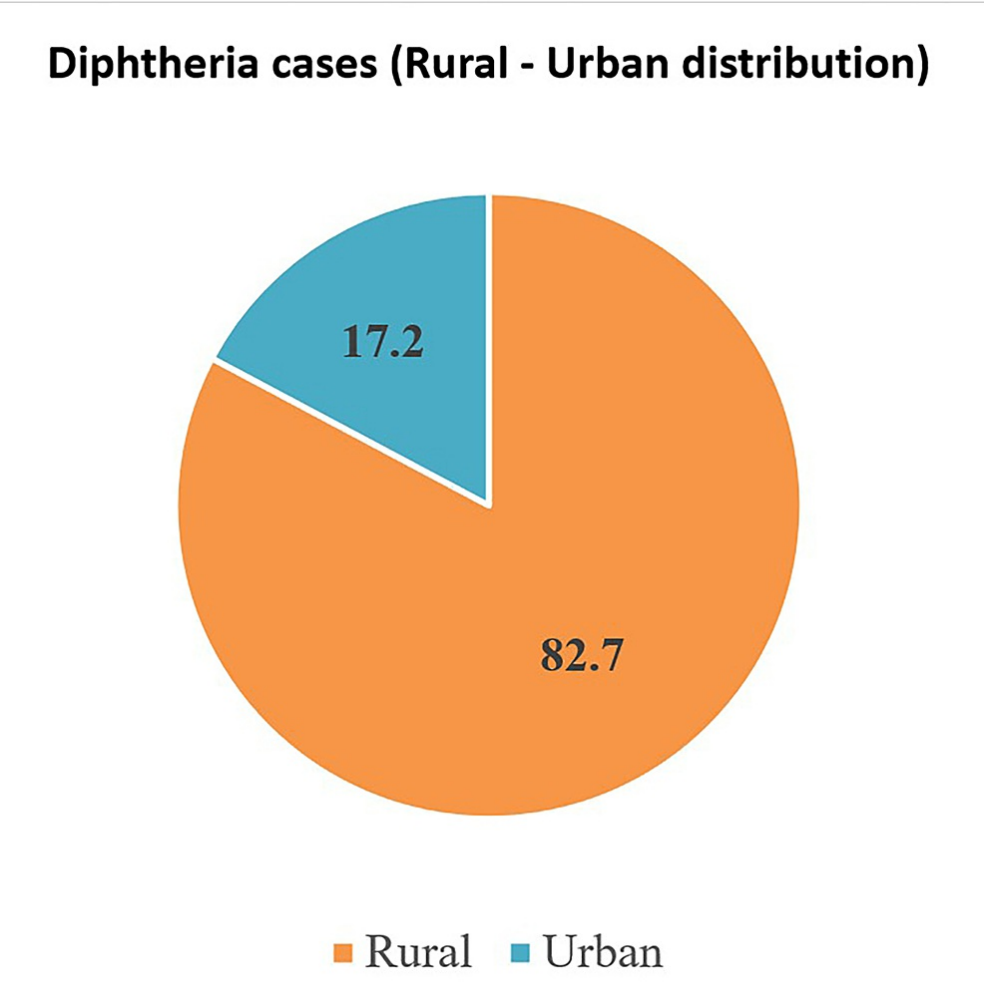
Rural-urban distribution of diphtheria patients

Of the 255 patients, 190 (74.5%) cases were reported from the Banaskantha district by only three blocks (93 were reported from the Dhanera block, 59 from the Deesa block, and 38 from the Lakhni block). The remaining 131 patients were from the 14 districts and seven corporations of Gujarat state (Figure 2).

Time distribution

**Figure 3:**
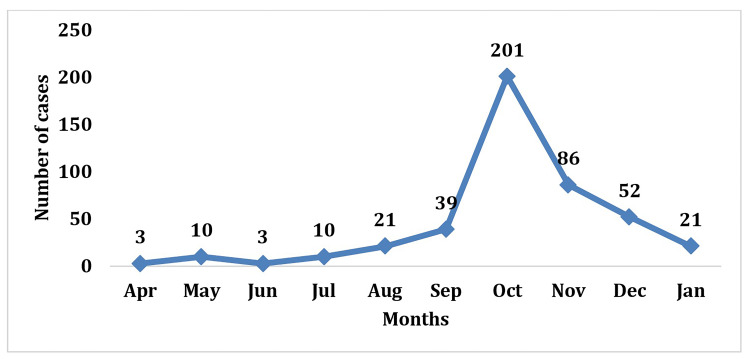
Month-wise occurrence of diphtheria cases in 2020-21 (n=446)

Month-wise distribution of the occurrence of diphtheria during the year shows that 339 (76%) patients were reported from September to December (Figure 3).

Immunization and survival status among the reported patients with diphtheria

The overall case-fatality ratio is 5.4% (24 deaths out of 446) among the reported diphtheria patients in the state during the study period. The number of patients who had not taken the DPT booster dose was 23 (96%), while 367 (87%) were alive but did not report the receipt of the DPT booster dose (Table 2). Here, the difference in frequency suggested the importance of the DPT booster dose in the case of mortality. However, there was no statistical significance in the association between DPT boosters dose and reported patients’ survival status.

**Table 2:** Immunization status and mortality (N=446)

DPT booster dose vaccination status					
Particulars	Death	Alive	Total	Chi-square p-value	Odds ratio
Received (n and %)	1 (4)	56 (13)	57 (13)	1.68 (0.194911)	0.2849
Not received (n and %)	23 (96)	367 (87)	390 (87)		
Total (n and %)	24 (100)	423 (100)	447 (100)		

## Discussion

Over the last two decades, the immunization coverage of DPT3 doses has substantially increased from 64.1 in 1998-1999 (NFHS 2) to 86.1 in 2019-2021 (NFHS 5) in India. This has significantly reduced the number of diphtheria cases. Although diphtheria cases have declined, it is still a major public health problem in most developing countries, where apart from immunization coverage, several other factors play an essential role in the occurrence of diphtheria. Of the 446 patients, the majority (255) were reported from the Banaskantha district, and 28 were reported from the Kutch district. The population distribution in the vast geography of Banaskantha and Kutch districts of Gujarat and the migratory population along the border of these districts may be the causes of the rise. All other districts had reported less than 20 cases, emphasizing the need to strengthen the surveillance system through regular monitoring and training of the reporting unit officer. The outbreak of the COVID-19 pandemic during 2020-2021 has impacted the reporting of diphtheria cases from the reporting unit.

It was observed that 76% (340) were in the age group of 5-14 years of age, of which 218 (49%) were in the 5-9 years and 122 (27%) in the 10-14 years of age group, signifying the prevalence of diphtheria in patients up to 14 years of age. Similar findings with the number of patients above 5 years of age were observed in other studies conducted in different world geographies: in central India (55%), East Kalimantan province in Indonesia (66.7 %), and 19 and 15 patients (maximum) reported in 2016 and 2017 in a study in Purwarkarta regency in Indonesia [6,10,11]. Myths and misconceptions on immunization, literacy of parents, migration, religion, and fear of side effects could be the various factors leading to a dropout of children [1]. Also, the reporting of diphtheria cases got impacted by the outbreak of COVID-19 during 2020-2021.

Three-fourths of patients (76%, 339 out of 446) were reported between October and December and 201 (45%) in October, suggesting that environmental and seasonal factors attributed to disease incidence patterns. Similar findings were also observed in the epidemiology of diphtheria in the Purwakarta regency of Indonesia [11] based on the retrospective study on reported diphtheria cases in the Rajkot district of Gujarat [7]. Further system side and demand side gaps mapping studies may be required to understand the critical factors triggering the occurrence of diphtheria between September and December.

Of 446 reported diphtheria patients in 2020-2021, 173 were immunized by the first pentavalent/DPT vaccine, 155 up to the second dose, 144 up to the third dose, 57 first booster dose, and 49 up to the second booster dose. Out of the 24 deaths, 23 were found in patients who were not vaccinated up to the first DPT booster dose.

Out of the 57 cases of diphtheria who completed the first DPT booster dose, only one death was reported, suggesting less fatality rate in the children vaccinated with the DPT vaccine corroborating with the findings of other studies [7,12]. No statistical significance was found in the association between the status of DPT boosters and deaths among the patients correlating [7] and contradicting [13,14] with the findings of another study.

The study was conducted based on secondary data from the government system of the state. Any shortcomings in reporting by the district’s team may impact the study findings. Detailed information on factors like the nutritional status of children and pre-existing diseases (other than the VPD) can provide further evidence related to diphtheria in children. Detailed information on various socio-demographics, clinical profiles, nutritional statuses, etc. should be collected using a pre-structured checklist to generate more evidence on factors affecting VPD mortality and morbidity. However, the study highlighted the various characteristics of diphtheria patients from secondary data in line with various other studies conducted across the globe. It has provided the learnings to strengthen the immunization and VPD surveillance system to minimize VPD case morbidity and mortality with geographic-specific action plans.

## Conclusions

Diphtheria occurs more commonly from September to December. The proportion of diphtheria was high in the 5-14 age group (76%), with slightly more diphtheria in males than females. The male-female ratio was 1.06 for all reported cases, while the male-female ratio was 1.16 among those who had not taken DPT3/pentavalent vaccine. The deaths were observed with incomplete DPT vaccine coverage including booster doses. Increased vaccination coverage and completing all doses of the DPT vaccine are crucial to avert deaths due to diphtheria.

The technology used to track individual children on their immunization status and their follow-up will help reduce the dropout and left-out cases and complete the full universal immunization schedule, which may be beneficial in preventing other VPDs too. In addition, an effective surveillance system and capacity building of the health workforce will aid in early disease detection and provide more information on the factors that led to disease occurrence for prompt action by the authority.
